# Supplementary material for: Moonlighting on the Fasciola hepatica tegument: Enolase, a glycolytic enzyme, interacts with the extracellular matrix and fibrinolytic system of the host
Source: PLoS Negl Trop Dis. 2024 Aug 30;18(8):e0012069. doi: 10.1371/journal.pntd.0012069 (PMC11392403; doi:10.1371/journal.pntd.0012069)
Supplement: S1 Fig — Sequence alignment of F. hepatica enolase (rFhENO) with three different O. aries enolase sequences which are alpha-enolase (accession number: XP_042113226), beta-enolase (accession number: XP_012040978), and gamma-enolase (accession number: XP_012030890). The table depicts the % identity and % similar positions of each O. aries enolase to FhENO. (DOCX) [file pntd.0012069.s001.docx]

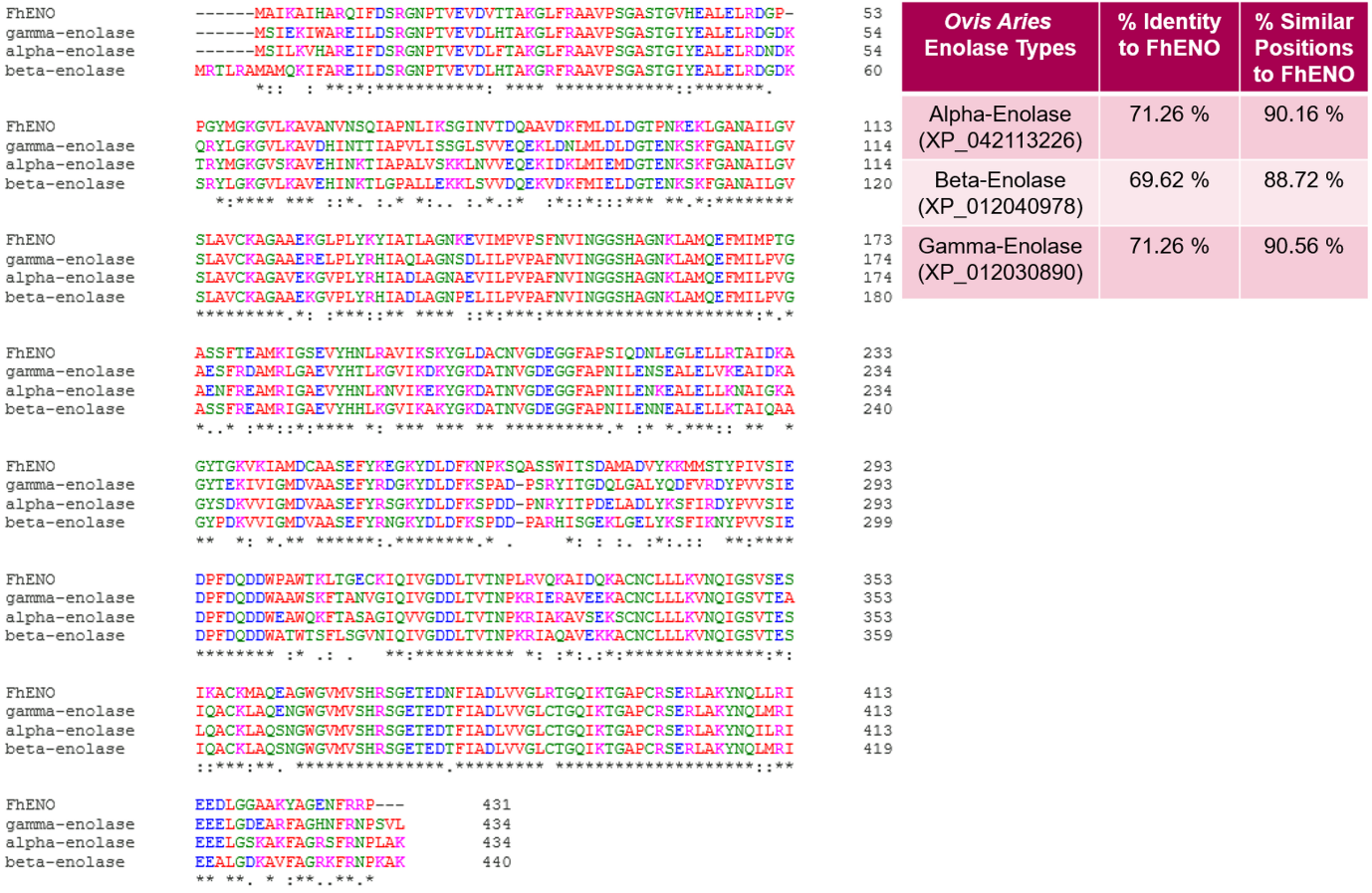


**Fig S1. Sequence alignment of *F. hepatica* enolase against three host *O. aries* enolases.** Sequence alignment of *F. hepatica* enolase (rFhENO) with three different *O. aries* enolase sequences which are alpha-enolase (accession number: XP_042113226), beta-enolase (accession number: XP_012040978), and gamma-enolase (accession number: XP_012030890). The table depicts the % identity and % similar positions of each *O. aries* enolase to FhENO.
