# Supplementary material for: Moonlighting on the Fasciola hepatica tegument: Enolase, a glycolytic enzyme, interacts with the extracellular matrix and fibrinolytic system of the host
Source: PLoS Negl Trop Dis. 2024 Aug 30;18(8):e0012069. doi: 10.1371/journal.pntd.0012069 (PMC11392403; doi:10.1371/journal.pntd.0012069)
Supplement: S2 Fig — (A) Maximum likelihood phylogram (unrooted) generated by PhyML based on the protein sequence Met1-Arg429 (Fh_eno_1 nomenclature), from representative enolase sequences from 14 trematode species compared with host (human and sheep) sequences: Cs, Clonorchis sinensis—Cs_eno_1: CSKR_100503, Cs_eno_2: CSKR_104864; Dd, Dicrocoelium dendriticum—Dd_eno_1: DDND1_54290; Ec, Echinostoma caproni—Ec_eno_1: ECPE_0000965901; Fh, Fasciola hepatica—Fh_eno_1: FhHiC23_g15231, Fh_eno_2: FhHiC23_g9518; Fg, Fasciola gigantica—Fg_eno_1: GWHPAZTT000715, Fg_eno_2: GWHPAZTT010334; Fb, Fasciolopsis buski—Fb_eno_1: FBUS_03870, Fb_eno_2: FBUS_04083; Ha, Heterobilharzia americana—Ha_eno_1: HAMR1_26230; Hs, Homo sapiens—Hs_alpha: NP_001419, Hs_beta: NP_001361452, Hs_gamma: NP_001966; Oa, Ovis aries—Oa_alpha: XP_042113226, Oa_beta: A0A6P3TWQ3, Oa_gamma: W5P5C0; Ov, Opisthorchis viverrini—Ov_eno_1: T265_10327, Ov_eno_2: T265_03262; Ph, Paragonimus heterotremus—Ph_eno_1: PHET_04041, Ph_eno_2: PHET_02710, Ph_eno_3: PHET_06263; Sb, Schistosoma bovis—Sb_eno_1: ACC78611; Sh, Schistosoma haematobium—Sh_eno_1: MS3_00003995; Sj, Schistosoma japonicum—Sj_eno_1: ACV41761; Sm, Schistosoma mansoni—Sm_eno_1: Smp_024110; Tr, Trichobilharzia regenti—Tr_eno_1: TREG1_20000. Bootstrap support values (1000 iterations) are shown at each node. (B) Clustal Omega alignment of the enolase sequences from the class Trematoda, compared to sequences from human and sheep as representative host species for Fasciola hepatica. Accession number/protein identifiers used as above. (DOCX) [file pntd.0012069.s002.docx]

**A**


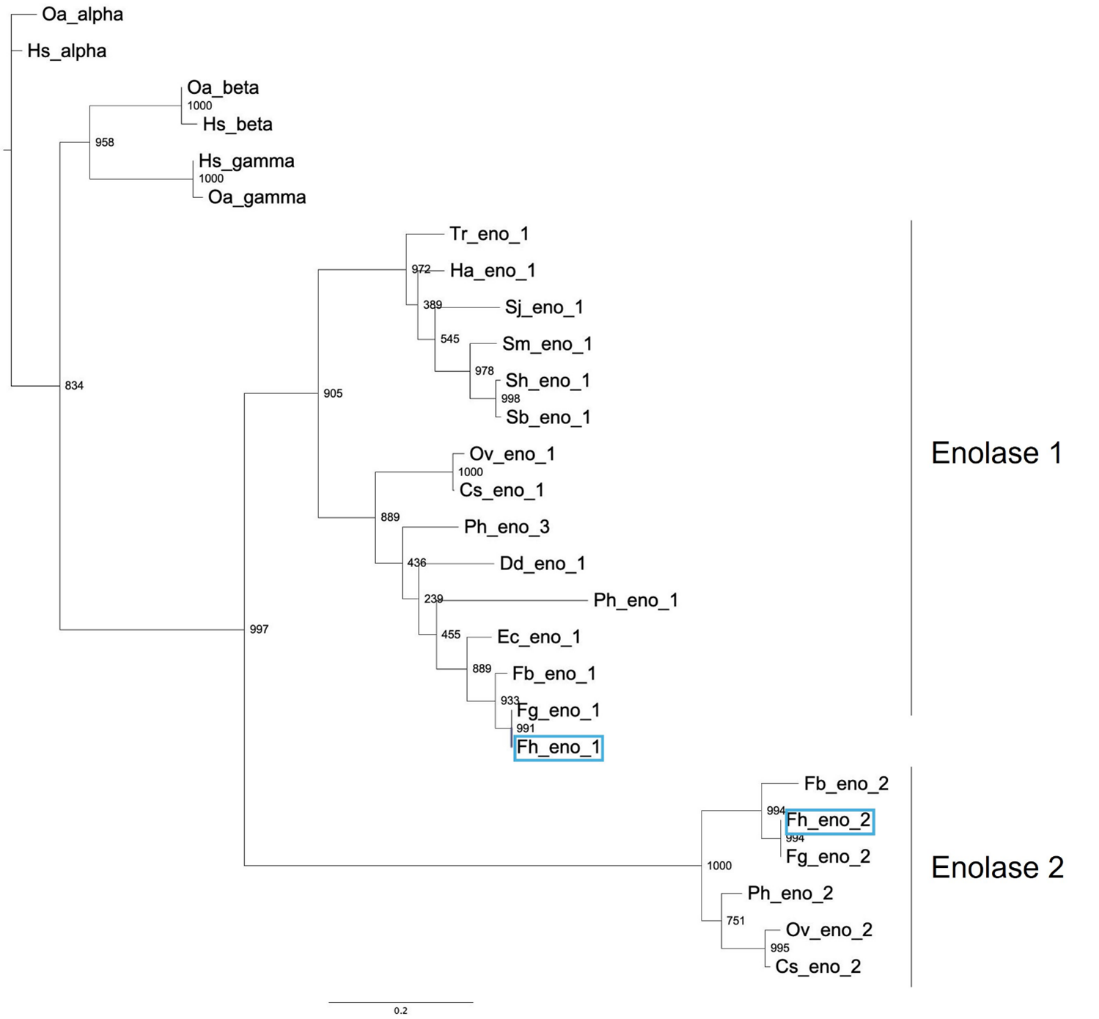


**B**

Ph_eno_2 MSILKITARQILDSRGYPTVEVDLVTRKGLFRAAVPSGASTGIYEANEMRDN-GKEYQGK 59

Cs_eno_2 MSILKITGRQILDSRGYPTVEVDLVTRKGLFRAAVPSGASTGIHEANEMRDS-GPEYHGK 59

Ov_eno_2 MSILKITGRQILDSRGYPTVEVDLVTRKGLFRAAVPSGASTGIHEANEMRDS-GPEYHGK 59

Fh_eno_2 MSILKITARQILDSRGFPTVEVDLVTHKGLFRAAVPSGASTGIYEANEMRDN-TREYQGK 59

Fg_eno_2 MSILKITARQILDSRGFPTVEVDLVTHKGLFRAAVPSGASTGIYEANEMRDN-TREYQGK 59

Fb_eno_2 MSILKITARQILDSRGFPTVEVDLVTHKGLFRAAVPSGASTGIYEANEMRDN-TREYQGK 59

Oa_gamma MSIEKIWAREILDSRGNPTVEVDLHTAKGLFRAAVPSGASTGIYEALELRDGDKQRYLGK 60

Hs_gamma MSIEKIWAREILDSRGNPTVEVDLYTAKGLFRAAVPSGASTGIYEALELRDGDKQRYLGK 60

Oa_beta MAMQKIFAREILDSRGNPTVEVDLHTAKGRFRAAVPSGASTGIYEALELRDGDKSRYLGK 60

Hs_beta MAMQKIFAREILDSRGNPTVEVDLHTAKGRFRAAVPSGASTGIYEALELRDGDKGRYLGK 60

Oa_alpha MSILKVHAREIFDSRGNPTVEVDLFTAKGLFRAAVPSGASTGIYEALELRDNDKTRYMGK 60

Hs_alpha MSILKIHAREIFDSRGNPTVEVDLFTSKGLFRAAVPSGASTGIYEALELRDNDKTRYMGK 60

Sj_eno_1 MAIIAIHARQIFDSRGNPTVEVDLKTAKGLFRAAVPSGASTGVHEALELRDTNSKAYMCK 60

Sm_eno_1 MSILTIHARQIFDSRGNPTVEVDLKTSKGLFRAAVPSGASTGVHEALELRDTNSKAYMKK 60

Sb_eno_1 MSIIAIHARQIFDSRGNPTVEVDLKTSKGLFRAAVPSGASTGVHEALELRDTKSKAYMGK 60

Sh_eno_1 MSIISIHARQIFDSRGNPTVEVDLKTSKGLFRAAVPSGASTGVHEALELRDTKSKAYMGK 60

Ha_eno_1 MTIHSIHARQIFDSRGNPTVEVDLKTDKGLFRAAVPSGASTGVHEALELRDTNAKAYMRK 60

Tr_eno_1 MAIKSIHARQIFDSRGNPTVEVDLKTDKGLFRAAVPSGASTGVHEALELRDTSEKAYMRK 60

Ph_eno_1 MVIHEIHARQIFDSRGNPTVEVDVKTANGLFRAAVPSGASTGIHEALELRDG-PPGYMGK 59

Cs_eno_1 MAIKAIHARQIFDSRGNPTVEVDVTTDKGLFRAAVPSGASTGVHEALELRDG-GKVYMGK 59

Ov_eno_1 MAIKAIHARQIFDSRGNPTVEVDLTTDKGLFRAAVPSGASTGVHEALELRDG-GKVYMGK 59

Dd_eno_1 MTIKKIHARQIYDSRGNPTVEVDVTTDKGLFRSAVPSGASTGVHEALELRDG-PPGHMGK 59

Ph_eno_3 MAIKSVHARQIFDSRGNPTIEVDLITDKGLFRAAVPSGASTGVHEALELRDG-PPGYMGK 59

Ec_eno_1 MAIKSIHARQIFDSRGNPTVEVDVTTAKGLFRAAVPSGASTGVHEALELRDG-PSGYMGK 59

Fh_eno_1 MAIKAIHARQIFDSRGNPTVEVDVTTAKGLFRAAVPSGASTGVHEALELRDG-PPGYMGK 59

Fg_eno_1 MAIKAIHARQIFDSRGNPTVEVDVTTAKGLFRAAVPSGASTGVHEALELRDG-PPGYMGK 59

Fb_eno_1 MAIKAIHARQIFDSRGNPTVEVDVTTAKGLFRAAVPSGASTGVHEALELRDG-PPGYMGK 59

* : : .*:* **** **:***: * :* **:*********::** *:** : *

Ph_eno_2 GVMKAVNNINKIIAPALIRKNLCVTEQHAIDEFMVRELDGTTNKTHLGANAILAVSLAVL 119

Cs_eno_2 GVMKAVNNVNKIIAPALIKKNLCVTEQHAIDEFMVTELDGTKNKTHLGANAILAVSLAVL 119

Ov_eno_2 GVMKAVNNVNKIIAPALIKKNLCVTEQHAIDEFMVTELDGTTNKTHLGANAILAVSLAVL 119

Fh_eno_2 GVMKAVNNVNKIIAPALIRKNLCVTEQHAIDEFMVKELDGTSNKTHLGANAILAVSLAVL 119

Fg_eno_2 GVMKAVNNVNKIIAPALIRKNLCVTEQHAIDEFMVKELDGTSNKTHLGANAILAVSLAVL 119

Fb_eno_2 GVMKAVNNVNRIIAPALISKNLCVTEQHAIDEFMVNELDGTTNKTHLGANAILAVSLAVL 119

Oa_gamma GVLKAVDHINTTIAPVLISSGLSVVEQEKLDNLML-DLDGTENKSKFGANAILGVSLAVC 119

Hs_gamma GVLKAVDHINSTIAPALISSGLSVVEQEKLDNLML-ELDGTENKSKFGANAILGVSLAVC 119

Oa_beta GVLKAVEHINKTLGPALLEKKLSVVDQEKVDKFMI-ELDGTENKSKFGANAILGVSLAVC 119

Hs_beta GVLKAVENINNTLGPALLQKKLSVVDQEKVDKFMI-ELDGTENKSKFGANAILGVSLAVC 119

Oa_alpha GVSKAVEHINKTIAPALVSKKLNVVEQEKIDKLMI-EMDGTENKSKFGANAILGVSLAVC 119

Hs_alpha GVSKAVEHINKTIAPALVSKKLNVTEQEKIDKLMI-EMDGTENKSKFGANAILGVSLAVC 119

Sj_eno_1 GVLTAVSNVNNIIAPALLKKQIPVTNQSEVDQFMI-ELDGKENKGNLGANAILGVSLAVC 119

Sm_eno_1 GVLTAVSNVNKIIAPALINKNIPVTNQAAIDKYMI-DLDGTENKEKLGANAILGVSLAVC 119

Sb_eno_1 GVLTAVSNVNTIIAPALIQKNIPVTDQAAIDRFMI-ELDGTENKEKLGANAILGVSLAVC 119

Sh_eno_1 GVLTAVSNVNTTIAPALIQKNIPVTDQAAIDRFMI-DLDGTENKEKLGANAILGVSLAVC 119

Ha_eno_1 GVLTAVNNVNKIIAPALLSKQIPVTDQAAIDKLMI-DLDGTENKEKLGANAILGVSLAVC 119

Tr_eno_1 GVLTAVNNVNKIIAPALLKENIPVTDQTTIDNYML-KLDGTENKGKLGANAILGVSLAVC 119

Ph_eno_1 SVEKAVANVNVHIAPNLLKCNIDVTDQAVIDRFML-SLDGTPNKEKLGANAILGVSLAMC 118

Cs_eno_1 GVMKAVENVNKIIAPALLKCGISVENQAEIDKIML-QLDGTANKEKLGANAILGVSLAVC 118

Ov_eno_1 GVTKAVENVNKIIAPALLKCGISVENQGEIDKIML-QLDGTANKEKLGANAILGVSLAVC 118

Dd_eno_1 GVMKAVDNVNKTIAPALLKSNIAVTDQTAIDKLML-SLDGTANKDKLGANAILGVSLAVC 118

Ph_eno_3 GVLKAVENVNSQIAPALLKANLPVTDQTAIDKLML-SLDGTANKGKLGANAILGVSLAVC 118

Ec_eno_1 GVLKAVSNVNNQIAPGLLKCGIPVTDQAGIDNFML-QLDGTPNKEKLGANAILGVSLAVC 118

Fh_eno_1 GVLKAVANVNSQIAPNLIKSGINVTDQAAVDKFML-DLDGTPNKEKLGANAILGVSLAVC 118

Fg_eno_1 GVLKAVANVNSQIAPNLIKSGINVTDQAAVDKFML-DLDGTPNKEKLGANAILGVSLAVC 118

Fb_eno_1 GVLKAVANVNSQIGPNLIKSGIHVTDQAAVDKFML-DLDGTPNKEKLGANAILGVSLAVC 118

.* .** ::* :.* *: : * :* :*. *: .:**. ** ::******.****:

Ph_eno_2 KAGAAEKNLPVYRWVANLAGNRTVTLPVPAFNVLNGGKHAGNRLAFQEFMILPVGAKSFA 179

Cs_eno_2 KAGAAEKNLPVYRWVANLAGNEKVTLPVPAFNVLNGGKHAGNRLAFQEFMILPVGAKSFA 179

Ov_eno_2 KAGAAEKNLPVYRWVANLAGNEKVTLPVPAFNVLNGGKHAGNRLAFQEFMILPVGAKSFA 179

Fh_eno_2 KAGAAEKNLPVYRWVANLAGNRTMSLPVPAFNVLNGGKHAGNSLAFQEFMILPVGAKSFA 179

Fg_eno_2 KAGAAEKNLPVYRWVANLAGNRTMSLPVPAFNVLNGGKHAGNSLAFQEFMILPVGAKSFA 179

Fb_eno_2 KAGAAEKNLPVYRWVANLAGNHNICLPVPAFNVLNGGKHAGNSLAFQEFMILPVGAKSFA 179

Oa_gamma KAGAAERELPLYRHIAQLAGNSDLILPVPAFNVINGGSHAGNKLAMQEFMILPVGAESFR 179

Hs_gamma KAGAAERELPLYRHIAQLAGNSDLILPVPAFNVINGGSHAGNKLAMQEFMILPVGAESFR 179

Oa_beta KAGAAEKGVPLYRHIADLAGNPELILPVPAFNVINGGSHAGNKLAMQEFMILPVGASSFR 179

Hs_beta KAGAAEKGVPLYRHIADLAGNPDLILPVPAFNVINGGSHAGNKLAMQEFMILPVGASSFK 179

Oa_alpha KAGAVEKGVPLYRHIADLAGNAEVILPVPAFNVINGGSHAGNKLAMQEFMILPVGAENFR 179

Hs_alpha KAGAVEKGVPLYRHIADLAGNSEVILPVPAFNVINGGSHAGNKLAMQEFMILPVGAANFR 179

Sj_eno_1 KAGAAELNLPLYRYIAKLAGHKDVIMPVPAFNVINGGSHAGNKLAMQEFMILPTGASSFT 179

Sm_eno_1 KAGAAEAGLPLYRYIARLAGHEDVIMPVPAFNVINGGSHAGNKLAMQEFMILPTGASSFT 179

Sb_eno_1 KAGAAEAGLPLYRYIAKLAGHENVIMPVPAFNVINGGSHAGNKLAMQEFMILPTGASSFT 179

Sh_eno_1 KAGAAEAGLPLYRYIAKLAGHENVIMPVPAFNVINGGSHAGNKLAMQEFMILPTGASSFT 179

Ha_eno_1 KAGAAEAGLPLYRYIAKLAGNTDVIMPVPAFNVINGGSHAGNKLAMQEFMILPTGASSFT 179

Tr_eno_1 KAGAAECGLPLYRYIAKLAGHSDVIMPVPAFNVINGGSHAGNKLAMQEFMILPTGASSFT 179

Ph_eno_1 KAGAAEMKMPLYQYIANMAGNRDVVLPVPAFNVINGGSHAGNKLAMQEFMIMPTGATSFK 178

Cs_eno_1 KAGAAQRNLPLYRHIASLAGNNDVILPVPAFNVINGGSHAGNKLAMQEFMIMPTGATSFK 178

Ov_eno_1 KAGAAQRNLPLYRHIASLAGNNDVILPVPAFNVINGGSHAGNKLAMQEFMIMPTGATSFK 178

Dd_eno_1 RAGAAEKGVPLYKYIASLAGNDQVILPVPSFNVINGGSHAGNKLAMQEFMIMPTGATSFK 178

Ph_eno_3 KAGAAEKGVPLYRYIASLAGNNDVVLPVPSFNVINGGSHAGNKLAMQEFMIMPTGAGSFK 178

Ec_eno_1 KAGAAEKGLPLYKYIASLAGNNDVVMPVPSFNVINGGSHAGNKLAMQEFMIMPTGASSFA 178

Fh_eno_1 KAGAAEKGLPLYKYIATLAGNKEVIMPVPSFNVINGGSHAGNKLAMQEFMIMPTGASSFT 178

Fg_eno_1 KAGAAEKGLPLYKYIATLAGNKEVIMPVPSFNVINGGSHAGNKLAMQEFMIMPTGASSFT 178

Fb_eno_1 KAGAAEKGLPLYKYIASLAGNNEVIMPVPSFNVINGGSHAGNKLAMQEFMIMPTGASSFS 178

:***.: :*:*: :* :**: : :***:***:***.**** **:*****:*.** .*

Ph_eno_2 EAVRMGSETYHCLREILKKKYGLNACNVGDEGGFAPNISTPSEALDLLVDAISNAGYVGK 239

Cs_eno_2 EAVRMGSETYHCLRNIIKAKYGLDACNVGDEGGFAPNISTPHDALDLLVDAISNAGYVGK 239

Ov_eno_2 EAVRMGSETYHCLRNIIKTKYGLDACNVGDEGGFAPNISTPHDALDLLVDAISNAGYAGK 239

Fh_eno_2 EALRMGSETYHCLRGIIKKKYGLDACNVGDEGGFAPNISTPVEALDLLVDAIAAAGFVGK 239

Fg_eno_2 EALRMGSETYHCLRGIIKKKYGLDACNVGDEGGFAPNISTPVEALDLLVDAIAAAGFVGK 239

Fb_eno_2 EALRMGSETYHCLKGIIKKKYGLDACNVGDEGGFAPNISTPAEALDMLVEAISVAGYVGK 239

Oa_gamma DAMRLGAEVYHTLKGVIKDKYGKDATNVGDEGGFAPNILENSEALELVKEAIDKAGYTEK 239

Hs_gamma DAMRLGAEVYHTLKGVIKDKYGKDATNVGDEGGFAPNILENSEALELVKEAIDKAGYTEK 239

Oa_beta EAMRIGAEVYHHLKGVIKAKYGKDATNVGDEGGFAPNILENNEALELLKTAIQAAGYPDK 239

Hs_beta EAMRIGAEVYHHLKGVIKAKYGKDATNVGDEGGFAPNILENNEALELLKTAIQAAGYPDK 239

Oa_alpha EAMRIGAEVYHNLKNVIKEKYGKDATNVGDEGGFAPNILENKEALELLKNAIGKAGYSDK 239

Hs_alpha EAMRIGAEVYHNLKNVIKEKYGKDATNVGDEGGFAPNILENKEGLELLKTAIGKAGYTDK 239

Sj_eno_1 EAMQMGSEVYHNLKAVIKREFGLDACNVGDEGGFAPNIQDNMKGLQLLEEAIKIAGYTGK 239

Sm_eno_1 EAMQIGTEVYHNLKAVIKREYGLDACNVGDEGGFAPNIQDNMKGLQLLEEAIKIAGYTGK 239

Sb_eno_1 EAMKIGSEVYHNLKAVIKREYGLDACNVGDEGGFAPNIQDNMKGLQLLEEAIKIAGYTGK 239

Sh_eno_1 EAMKIGSEVYHNLKAVIKREYGLDACNVGDEGGFAPNIQDNMKGLQLLEEAIKIAGYTGK 239

Ha_eno_1 EAMQIGTEVYHNLKAVIKRDYGLDACNVGDEGGFAPNIQDNMKGLQLLEEAIKIAGYSGK 239

Tr_eno_1 EAMRIGTEVYHNLKAVIKRDYGLDACNVGDEGGFAPNIQDNMKGLQLLEEAIKIAGYAGK 239

Ph_eno_1 EAMKMGSEVYHTLRSVIKSKYGLDACNVGDEGGFAPGIQDNMEGLDLLNTAIAKAGYTGR 238

Cs_eno_1 EAMQMGSEVYHNLKSVIKAKYGLDACNVGDEGGFAPNIQDNMEGLQLLNEAIAKAGYTGK 238

Ov_eno_1 EAMQMGSEVYHNLKSVIKAKYGLDACNVGDEGGFAPNIQDNMEGLQLLSEAIAKAGYTGK 238

Dd_eno_1 EAMKMGSEVYHNLRAVIKEKYGLDACNVGDEGGFAPNIQDNMEGLELLRTAIEKAHYTGK 238

Ph_eno_3 EAMKIGVEVYHNLKAVIKAKYGLDACNVGDEGGFAPNIQDNMEGLELLRVAIEKAGYTGK 238

Ec_eno_1 EAMKIGSEVYHHLKAVIKGKYGLDACNVGDEGGFAPNIQDNLEGLELLRTAIEKAGYTGK 238

Fh_eno_1 EAMKIGSEVYHNLRAVIKSKYGLDACNVGDEGGFAPSIQDNLEGLELLRTAIDKAGYTGK 238

Fg_eno_1 EAMKIGSEVYHNLRAVIKSKYGLDACNVGDEGGFAPSIQDNLEGLELLRTAIDKAGYTGK 238

Fb_eno_1 EAMKIGSEVYHNLRAVIKNKYGLDACNVGDEGGFAPSIQDNLEGLELLRTAIDKAGYTGK 238

:*:::* *.** *: ::* .:* :* **********.* ..*::: ** * : :

Ph_eno_2 IVIGMDVASSEMWIKGGKYNLNFKDPRQEPNQWISGDKLLDTYTSLLATYPIVTIEDPFD 299

Cs_eno_2 ILIGMDVASSEMYTTGGKYNMNFKDARQDPNQLISGDKLLDTYISLLSHYPIVSIEDPFD 299

Ov_eno_2 ILIGMDVASSEMYTTGGKYNLNFKDARQDPNQLISGDKLLDIYVGLLGHYPIVSIEDPFD 299

Fh_eno_2 IVIGMDVASSEMYIKGGKYDLNFKDTQTATRDALSGDKLLETYLTLVSRYPIVSIEDPFD 299

Fg_eno_2 IVIGMDVASSEMYIKGGKYDLNFKDTQTATRDALSGDKLLETYLTLVSRYPIVSIEDPFD 299

Fb_eno_2 IVIGMDVASSEMYVKGGKYDMNFKDTQTTSREVLTGDKLLEYYLNLVSHYPIVSIEDPFD 299

Oa_gamma IVIGMDVAASEFY-RDGKYDLDFKSPAD-PSRYITGDQLGALYQDFVRDYPVVSIEDPFD 297

Hs_gamma IVIGMDVAASEFY-RDGKYDLDFKSPTD-PSRYITGDQLGALYQDFVRDYPVVSIEDPFD 297

Oa_beta VVIGMDVAASEFY-RNGKYDLDFKSPDD-PARHISGEKLGELYKSFIKNYPVVSIEDPFD 297

Hs_beta VVIGMDVAASEFY-RNGKYDLDFKSPDD-PARHITGEKLGELYKSFIKNYPVVSIEDPFD 297

Oa_alpha VVIGMDVAASEFY-RSGKYDLDFKSPDD-PNRYITPDELADLYKSFIRDYPVVSIEDPFD 297

Hs_alpha VVIGMDVAASEFF-RSGKYDLDFKSPDD-PSRYISPDQLADLYKSFIKDYPVVSIEDPFD 297

Sj_eno_1 VEIGMDCAASEYY-KKGKYDLDFKNPQSAESHWLSPDEMANVYKEMIQKYPIVSIEDPFD 298

Sm_eno_1 VEIGMDCAASEFH-KNGKYDLDFKNPHSAESTWLSPDAMANMYKQMISKFPIVSIEDPFD 298

Sb_eno_1 KEIGMDCAASEFH-KNGKYDLDFKNPHSAESAWLSPDAMTNVYKEMISKYPIVSIEDPVD 298

Sh_eno_1 VEIGMDCAASEFH-KNGKYDLDFKNPHSAESAWLSPDAMTNVYKEMISKYPIVSIEDPVD 298

Ha_eno_1 VEIGMDCAASEYY-KNGKYDLDFKNPQSPENTWLTPDAMADVYKEMIDKYPIVSIEDPFD 298

Tr_eno_1 VEIGMDCAASEYY-KDGKYDLDFKNPQSQPSSWLSPDAMAGVYKEMINKYPIVSIEDPFD 298

Ph_eno_1 IKIAMDCAASEFY-KQGKYDLDFKNPNSRPNTWLTSDDMEAVYKKMISNYPIVSIEDPFD 297

Cs_eno_1 VKIAMDSAASEFH-KDGKYDLDFKNPNSPPNTWISSDALGDVYKSMISKYPIVSIEDPFD 297

Ov_eno_1 VKIAMDSAASEFH-KNGKYDLDFKNPNSPPNTWISSDALGDVYKSMIGKYPIVSIEDPFD 297

Dd_eno_1 IKIAMDCAASEFH-KQGKYDLDFKNVKSAPDSWISSESLADVYKKMMSTYPIVSIEDPFD 297

Ph_eno_3 VKIAMDSAASEFY-KEGKYDLDFKNPNSPPSTWISSDALGDVYKKMINNYPIVSIEDPFD 297

Ec_eno_1 VKIAMDSAASEFY-KEGKYDLDFKNPKSPASSWISSDAMADVYKKMMSTYPIVSIEDPFD 297

Fh_eno_1 VKIAMDCAASEFY-KEGKYDLDFKNPKSQASSWITSDAMADVYKKMMSTYPIVSIEDPFD 297

Fg_eno_1 VKIAMDCAASEFY-KEGKYDLDFKNPKSQASSWITSDAMADVYKKMMSTYPIVSIEDPFD 297

Fb_eno_1 VKIAMDCAASEFY-KEGKYDLDFKNPKSPASSWITSDAMADVYKKMMSTYPIVSIEDPFD 297

*.** *:** ***:::**. :: : : * :: :*:*:****.*

Ph_eno_2 QDDWEHWIKFRNRSKVQIVGDDLLVTNPERVRKAIEVGACNALLLKVNQIGSFTEALEAC 359

Cs_eno_2 QDDWEHWIKFRSRSKVQIVGDDLLVTNPERVRKAIELGACNALLLKVNQIGSFTEALEAC 359

Ov_eno_2 QDDWEHWIKFRSRSKVQIVGDDLLVTNPQRVRRAIELGACNALLLKVNQIGSFTEALEAC 359

Fh_eno_2 QDDWEHWIKFRSNSNIQIVGDDLLVTNPARVQRAIEVGACNALLLKVNQIGSFTEALQAC 359

Fg_eno_2 QDDWEHWIKFRSNSNIQIVGDDLLVTNPARVQRAIEVGACNALLLKVNQIGSFTEALQAC 359

Fb_eno_2 QDDWDHWIKFRSNSNIQIVGDDLLVTNPERVKRAIEVGACNALLLKVNQIGSFTEALRAC 359

Oa_gamma QDDWAAWSKFTANVGIQIVGDDLTVTNPKRIERAVEEKACNCLLLKVNQIGSVTEAIQAC 357

Hs_gamma QDDWAAWSKFTANVGIQIVGDDLTVTNPKRIERAVEEKACNCLLLKVNQIGSVTEAIQAC 357

Oa_beta QDDWATWTSFLSGVNIQIVGDDLTVTNPKRIAQAVEKKACNCLLLKVNQIGSVTESIQAC 357

Hs_beta QDDWATWTSFLSGVNIQIVGDDLTVTNPKRIAQAVEKKACNCLLLKVNQIGSVTESIQAC 357

Oa_alpha QDDWEAWQKFTASAGIQVVGDDLTVTNPKRIAKAVSEKSCNCLLLKVNQIGSVTESLQAC 357

Hs_alpha QDDWGAWQKFTASAGIQVVGDDLTVTNPKRIAKAVNEKSCNCLLLKVNQIGSVTESLQAC 357

Sj_eno_1 QDDWDAWPKLTASTNIQIVGDDLTVTNPKRIEKAIKVKACNCLLLKVNQIGSITESIEAC 358

Sm_eno_1 QDDWETWPKLTSSTNIQIVGDDLTVTNPKRIKQAIASKACNCLLLKVNQIGSLTESIEAC 358

Sb_eno_1 QDDWETWPKLTASTNIQIVGDDLTVTNPKRIKKAISSKACNCLLLKVNQIGSLTESIEAC 358

Sh_eno_1 QDDWETWPKLTASTNIQIVGDDLTVTNPKRIKKAISSKACNCLLLKVNQIGSLTESIEAC 358

Ha_eno_1 QDDWEAWPKLTASTHIQIVGDDLTVTNPKRIQKAIGAKACNCLLLKVNQIGSITESIEAC 358

Tr_eno_1 QDDWEAWPKLTASTQIQIVGDDLTVTNPKRIQKAIEAKACNCLLLKVNQIGSITESIEAC 358

Ph_eno_1 QDDWDAWAKLTASCNIQIVGDDLTVTNPVRVQQAIDRRACNCLLLKVNQIGTVTEAVRAC 357

Cs_eno_1 QDDWGAFAKLTGETSIQIVGDDLTVTNPIRVQEAINKKACNCLLLKVNQIGSVSESIQAC 357

Ov_eno_1 QDDWGAFAKLTGETSIQIVGDDLTVTNPVRVQEAINKKACNCLLLKVNQIGSVSESIQAC 357

Dd_eno_1 QDDWAAWSKLTGSCDIQIVGDDLTVTNPIRVQQAIDKKACNCLLLKVNQIGSVTESIQAC 357

Ph_eno_3 QDDWGAWSKLTSDVSVQIVGDDLTVTNPIRVQKAIEQKACNCLLLKVNQIGSVTESIQAC 357

Ec_eno_1 QDDWPAWTKLTGECKIQIVGDDLTVTNPLRVQKAIDQKACNCLLLKVNQIGSVTESIQAC 357

Fh_eno_1 QDDWPAWTKLTGECKIQIVGDDLTVTNPLRVQKAIDQKACNCLLLKVNQIGSVSESIKAC 357

Fg_eno_1 QDDWPAWTKLTGECKIQIVGDDLTVTNPLRVQKAIDQKACNCLLLKVNQIGSVSESIKAC 357

Fb_eno_1 QDDWAAWTKLTGECNIQIVGDDLTVTNPLRVQKAIEQKACNCLLLKVNQIGSVSESIKAC 357

**** : .: :*:***** **** *: .*: :**.*********:.:*::.**

Ph_eno_2 QMAKRANWKVMVSHRSGETEDSTIADLAVGLNCGQIKTGAPCRSERLAKYNQLLRIEEEL 419

Cs_eno_2 QMAKRANWKVMVSHRSGETEDSTIADLAVGLNCGQIKTGAPCRSERLAKYNQLLRIEEEL 419

Ov_eno_2 QIAKRANWKVMVSHRSGETEDSTIADLAVGLNCGQIKTGAPCRSERLAKYNQLLRIEEEL 419

Fh_eno_2 QMATRANWKVMVSHRSGETEDCTIADIAVGLNCGQIKTGAPCRSERLAKYNQLLRIEEEL 419

Fg_eno_2 QMATRANWKVMVSHRSGETEDCTIADIAVGLNCGQIKTGAPCRSERLAKYNQLLRIEEEL 419

Fb_eno_2 QMATRANWKVMVSHRSGETEDCTIADIAVGLNCGQIKTGAPCRSERLAKYNQLLRIEEEL 419

Oa_gamma KLAQENGWGVMVSHRSGETEDTFIADLVVGLCTGQIKTGAPCRSERLAKYNQLMRIEEEL 417

Hs_gamma KLAQENGWGVMVSHRSGETEDTFIADLVVGLCTGQIKTGAPCRSERLAKYNQLMRIEEEL 417

Oa_beta KLAQSNGWGVMVSHRSGETEDTFIADLVVGLCTGQIKTGAPCRSERLAKYNQLMRIEEAL 417

Hs_beta KLAQSNGWGVMVSHRSGETEDTFIADLVVGLCTGQIKTGAPCRSERLAKYNQLMRIEEAL 417

Oa_alpha KLAQSNGWGVMVSHRSGETEDTFIADLVVGLCTGQIKTGAPCRSERLAKYNQILRIEEEL 417

Hs_alpha KLAQANGWGVMVSHRSGETEDTFIADLVVGLCTGQIKTGAPCRSERLAKYNQLLRIEEEL 417

Sj_eno_1 KMAQKAGWGVMVSHRSGETEDNFIADLVVGLCTGQIKTGAPCRSERLAKYNQLLRIEEEL 418

Sm_eno_1 KLAQDSGWGVMVSHRSGETEDTFIADLVVGLCTGQIKTGAPCRSDRLAKYNQLLRIEEEL 418

Sb_eno_1 KLAQNAGWGVMVSHRSGETEDTFIADLVVGLCTGQIKAGAPCRSDRLAKYNQLLRIEEEL 418

Sh_eno_1 KLAQNAGWGVMVSHRSGETEDTFIADLVVGLCTGQIKAGAPCRSDRLAKYNQLLRIEEEL 418

Ha_eno_1 RLAQKAGWGVMVSHRSGETEDNFIADLVVGLCTGQIKTGAPCRSERLAKYNQLLRIEEEL 418

Tr_eno_1 KLAQKAGWGVMVSHRSGETEDNFIADLVVGLCTGQIKTGAPCRSERLAKYNQLLRIEEEL 418

Ph_eno_1 KLAQSAGWSVMVSHRSGETEDNFIADLVVGLRTGQIKTGAPCRSERLAKYNQLLRIEENL 417

Cs_eno_1 KMAQGAGWGVMVSHRSGETEDNFIADLVVGLRTGQIKTGAPCRSERLSKYNQLLRIEEDL 417

Ov_eno_1 KMAQGAGWGVMVSHRSGETEDNFIADLVVGLRTGQIKTGAPCRSERLSKYNQLLRIEEDL 417

Dd_eno_1 KLAQSAGWGVMVSHRSGETEDNFIADLVVGLCTGQIKTGAPCRSERLAKYNQLLRIEEDL 417

Ph_eno_3 KLAQSAGWGVMVSHRSGETEDNFIADLVVGLRTGQIKTGAPCRSERLSKYNQLLRIEEDL 417

Ec_eno_1 KMAQSAGWGVMVSHRSGETEDNFIADLVVGLRTGQIKTGAPCRSERLAKYNQLLRIEEDL 417

Fh_eno_1 KMAQEAGWGVMVSHRSGETEDNFIADLVVGLRTGQIKTGAPCRSERLAKYNQLLRIEEDL 417

Fg_eno_1 KMAQEAGWGVMVSHRSGETEDNFIADLVVGLRTGQIKTGAPCRSERLAKYNQLLRIEEDL 417

Fb_eno_1 KMAQSAGWGVMVSHRSGETEDNFIADLVVGLRTGQIKTGAPCRSERLSKYNQLLRIEEDL 417

::* .* ************ ***:.*** ****:******:**:****::**** *

Ph_eno_2 GCHAAYAGNVFKQSRLC--- 436

Cs_eno_2 GCHAVYAGNVFKQSTLC--- 436

Ov_eno_2 GCHAVYAGNVFKQSTLC--- 436

Fh_eno_2 GCYASYAGNVFKPTSKYFT- 438

Fg_eno_2 GCYASYAGNVFKPTNKYFT- 438

Fb_eno_2 GCYASYAGNVFKTLHNQCRS 439

Oa_gamma GDEARFAGHNFRNPSVL--- 434

Hs_gamma GDEARFAGHNFRNPSVL--- 434

Oa_beta GDKAVFAGRKFRNPKAK--- 434

Hs_beta GDKAIFAGRKFRNPKAK--- 434

Oa_alpha GSKAKFAGRSFRNPLAK--- 434

Hs_alpha GSKAKFAGRNFRNPLAK--- 434

Sj_eno_1 GSTAKYAGKHFRHPQI---- 434

Sm_eno_1 GTAAKYAGKNFRHPKV---- 434

Sb_eno_1 GAAAKYAGKNFRHPKK---- 434

Sh_eno_1 GAGAKYAGKNFRHPKV---- 434

Ha_eno_1 GTAAKYAGKHFRHPK----- 433

Tr_eno_1 GAAAKYAGKNFRHPQ----- 433

Ph_eno_1 GNMARYAGEHFRRP------ 431

Cs_eno_1 GSSAKYAGEKFRKP------ 431

Ov_eno_1 GSSAKYAGEKFRKP------ 431

Dd_eno_1 GAAAKYAGEHFRRPLK---- 433

Ph_eno_3 GANAKYAGEHFRRP------ 431

Ec_eno_1 GSAAKYAGENFRRP------ 431

Fh_eno_1 GGAAKYAGENFRRP------ 431

Fg_eno_1 GGAAKYAGENFRRP------ 431

Fb_eno_1 GSAAKYAGEHFRRP------ 431

* * :**. *:

**Fig S2. Phylogenetic analysis of the relatedness of enolase proteins within the class Trematoda.** (A) Maximum likelihood phylogram (unrooted) generated by PhyML based on the protein sequence Met^1^-Arg^429^ (Fh_eno_1 nomenclature), from representative enolase sequences from 14 trematode species compared with host (human and sheep) sequences: Cs, *Clonorchis sinensis* - Cs_eno_1: CSKR_100503, Cs_eno_2: CSKR_104864; Dd, *Dicrocoelium dendriticum* - Dd_eno_1: DDND1_54290; Ec, *Echinostoma caproni* - Ec_eno_1: ECPE_0000965901; Fh, *Fasciola hepatica* - Fh_eno_1: FhHiC23_g15231, Fh_eno_2: FhHiC23_g9518; Fg, *Fasciola gigantica* - Fg_eno_1: GWHPAZTT000715, Fg_eno_2: GWHPAZTT010334; Fb, *Fasciolopsis buski* - Fb_eno_1: FBUS_03870, Fb_eno_2: FBUS_04083; Ha, *Heterobilharzia americana* - Ha_eno_1: HAMR1_26230; Hs, *Homo sapiens* - Hs_alpha: NP_001419, Hs_beta: NP_001361452, Hs_gamma: NP_001966; Oa, *Ovis aries* - Oa_alpha: XP_042113226, Oa_beta: A0A6P3TWQ3, Oa_gamma: W5P5C0; Ov, *Opisthorchis viverrini* - Ov_eno_1: T265_10327, Ov_eno_2: T265_03262; Ph, *Paragonimus heterotremus* - Ph_eno_1: PHET_04041, Ph_eno_2: PHET_02710, Ph_eno_3: PHET_06263; Sb, *Schistosoma bovis* - Sb_eno_1: ACC78611; Sh, *Schistosoma haematobium* - Sh_eno_1: MS3_00003995; Sj, *Schistosoma japonicum* - Sj_eno_1: ACV41761; Sm, *Schistosoma mansoni* - Sm_eno_1: Smp_024110; Tr, *Trichobilharzia regenti* - Tr_eno_1: TREG1_20000. Bootstrap support values (1000 iterations) are shown at each node. (B) Clustal Omega alignment of the enolase sequences from the class Trematoda, compared to sequences from human and sheep as representative host species for *Fasciola hepatica*. Accession number/protein identifiers used as above.
