# Supplementary material for: Moonlighting on the Fasciola hepatica tegument: Enolase, a glycolytic enzyme, interacts with the extracellular matrix and fibrinolytic system of the host
Source: PLoS Negl Trop Dis. 2024 Aug 30;18(8):e0012069. doi: 10.1371/journal.pntd.0012069 (PMC11392403; doi:10.1371/journal.pntd.0012069)
Supplement: S3 Fig — LC-MS was performed on the two bands detected by anti-rFhENO in lane 1 Fig 3A. (A) Peptides that were detected in the ~47 kDa band and matched areas of the rFhENO sequence are highlighted in blue. (B) Peptides that were detected in the ~37 kDa band and match rFhENO are also highlighted. (C) The peptides detected in both bands that match the rFhENO sequence are highlighted. (DOCX) [file pntd.0012069.s003.docx]

**(A) ~47 kDa Band: Peptides detected in the FhENO sequence:** 36.19% coverage

MAIKAIHARQIFDSRGNPTVEVDVTTAKGLFRAAVPSGASTGVHEALELRDGPPGYMGKGVLKAVANVNSQIAPNLIKSGINVTDQAAVDKFMLDLDGTPNKEKLGANAILGVSLAVCKAGAAEKGLPLYKYIATLAGNKEVIMPVPSFNVINGGSHAGNKLAMQEFMIMPTGASSFTEAMKIGSEVYHNLRAVIKSKYGLDACNVGDEGGFAPSIQDNLEGLELLRTAIDKAGYTGKVKIAMDCAASEFYKEGKYDLDFKNPKSQASSWITSDAMADVYKKMMSTYPIVSIEDPFDQDDWPAWTKLTGECKIQIVGDDLTVTNPLRVQKAIDQKACNCLLLKVNQIGSVSESIKACKMAQEAGWGVMVSHRSGETEDNFIADLVVGLRTGQIKTGAPCRSERLAKYNQLLRIEEDLGGAAKYAGENFRRP

**(B) ~35 kDa Band: Peptides detected in the FhENO sequence:** 48.26% coverage

MAIKAIHARQIFDSRGNPTVEVDVTTAKGLFRAAVPSGASTGVHEALELRDGPPGYMGKGVLKAVANVNSQIAPNLIKSGINVTDQAAVDKFMLDLDGTPNKEKLGANAILGVSLAVCKAGAAEKGLPLYKYIATLAGNKEVIMPVPSFNVINGGSHAGNKLAMQEFMIMPTGASSFTEAMKIGSEVYHNLRAVIKSKYGLDACNVGDEGGFAPSIQDNLEGLELLRTAIDKAGYTGKVKIAMDCAASEFYKEGKYDLDFKNPKSQASSWITSDAMADVYKKMMSTYPIVSIEDPFDQDDWPAWTKLTGECKIQIVGDDLTVTNPLRVQKAIDQKACNCLLLKVNQIGSVSESIKACKMAQEAGWGVMVSHRSGETEDNFIADLVVGLRTGQIKTGAPCRSERLAKYNQLLRIEEDLGGAAKYAGENFRRP

**(C) Peptides from both bands detected in the FhENO sequence**

MAIKAIHARQIFDSRGNPTVEVDVTTAKGLFRAAVPSGASTGVHEALELRDGPPGYMGKGVLKAVANVNSQIAPNLIKSGINVTDQAAVDKFMLDLDGTPNKEKLGANAILGVSLAVCKAGAAEKGLPLYKYIATLAGNKEVIMPVPSFNVINGGSHAGNKLAMQEFMIMPTGASSFTEAMKIGSEVYHNLRAVIKSKYGLDACNVGDEGGFAPSIQDNLEGLELLRTAIDKAGYTGKVKIAMDCAASEFYKEGKYDLDFKNPKSQASSWITSDAMADVYKKMMSTYPIVSIEDPFDQDDWPAWTKLTGECKIQIVGDDLTVTNPLRVQKAIDQKACNCLLLKVNQIGSVSESIKACKMAQEAGWGVMVSHRSGETEDNFIADLVVGLRTGQIKTGAPCRSERLAKYNQLLRIEEDLGGAAKYAGENFRRP

**Fig S3. Peptides detected by LC-MS mapped onto the rFhENO sequence.** LC-MS was performed on the two bands detected by anti-rFhENO in lane 1 Fig 3A. (A) Peptides that were detected in the ~47 kDa band and matched areas of the rFhENO sequence are highlighted in blue. (B) Peptides that were detected in the ~37 kDa band and match rFhENO are also highlighted. (C) The peptides detected in both bands that match the rFhENO sequence are highlighted.
