# Supplementary material for: Moonlighting on the Fasciola hepatica tegument: Enolase, a glycolytic enzyme, interacts with the extracellular matrix and fibrinolytic system of the host
Source: PLoS Negl Trop Dis. 2024 Aug 30;18(8):e0012069. doi: 10.1371/journal.pntd.0012069 (PMC11392403; doi:10.1371/journal.pntd.0012069)
Supplement: S4 Fig — The secondary structure of rFhENO was predicted using the Swiss-Model web server (http://swissmodel.expasy.org/) based on available X-ray crystallography models. The three-dimensional model of the molecule was visualized using RasMol application v. 2.7.5. Lysine residues of rFhENO, potential plasminogen-binding sites, were highlighted in red. (DOCX) [file pntd.0012069.s004.docx]

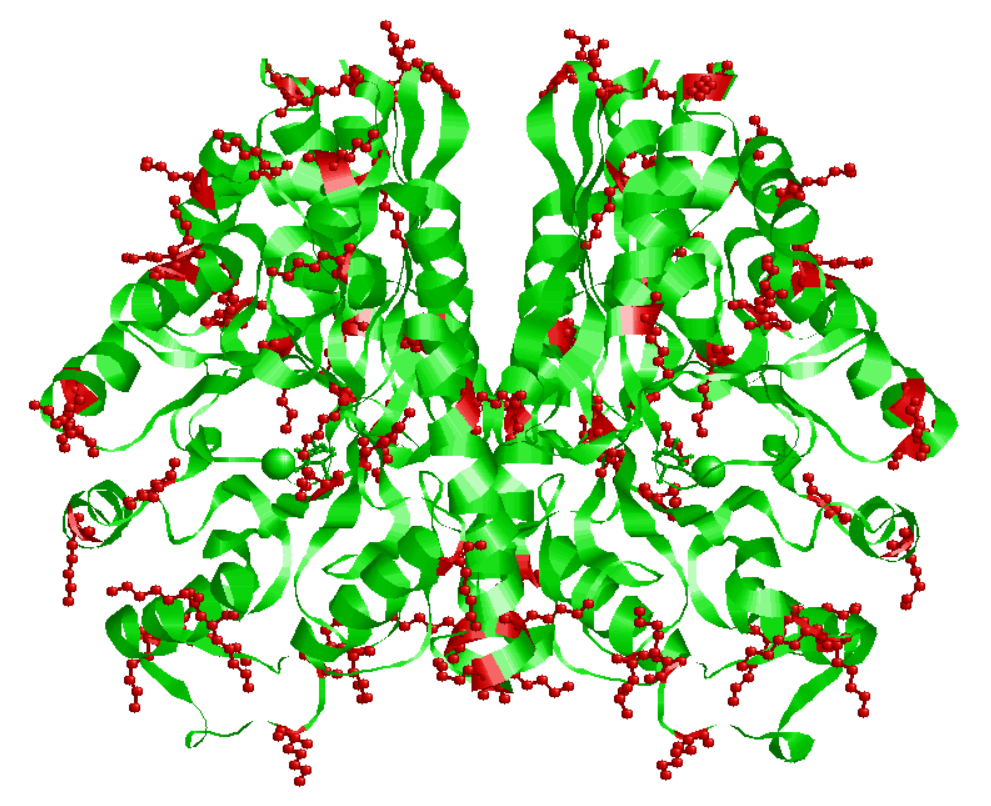


**Fig S4. Molecular modeling of rFhENO.** The secondary structure of rFhENO was predicted using the Swiss-Model web server (http://swissmodel.expasy.org/) based on available X-ray crystallography models. The three-dimensional model of the molecule was visualized using RasMol application v. 2.7.5. Lysine residues of rFhENO, potential plasminogen-binding sites, were highlighted in red.
